# Using Routine Data to Categorize Poor Control Diabetic Patients: An Application of Cluster Analysis Technique

**Published:** 2017-01

**Authors:** Farid ABOLHASSANI SHAHREZA, Narjes HAZAR

**Affiliations:** 1. Health Services Division, National Institute of Health Research, Tehran University of Medical Sciences, Tehran, Iran; 2. Deputy for Health Affairs, Shahid Sadoughi University of Medical Sciences, Yazd, Iran

**Keywords:** Diabetes, Poor control, Cluster analysis

## Abstract

**Background:**

Diabetes mellitus is one of the most important causes of morbidity and mortality all over the world. Chronic high blood glucose is the root cause of developing future micro and macro vascular complications. In current study, we analyzed poorly controlled patients’ information by clustering method in order to find more homogenous sub-groups of patients for planning for further interventions.

**Methods:**

This study was conducted based on medical records of diabetic patients admitted to 23 health care centers in four cities of Iran from 2010 to 2014. Demographic data and physical and biochemical measurements were extracted and analyzed using two-steps cluster analysis method.

**Results:**

Three distinct clusters were derived from 2087 eligible cases. Cluster 1 totally consisted of men without any apparent risk factors. Members of clusters 2 and 3 were illiterate women with obesity. Dyslipidemia was a prominent characteristic in members of cluster 2.

**Conclusion:**

Masculinity would play the main role in diabetes control. Meanwhile, aggregation of obesity, illiteracy and/or dyslipidemia in diabetic women predispose them to poor control condition. Based on these findings, we will be able to plan interventions that are more appropriate for identified groups.

## Introduction

The International Diabetes Federation (IDF) provided estimates of diabetes mellitus prevalence for 130 countries in 2013. According to this report, 4396000 Iranian peoples were diabetic in 2013 and the number would be increased to an estimated number of 8396000 in 2035 (1).

Diabetes mellitus is one of the most important causes of morbidity and mortality all over the world (2, 3). Long lasting hyperglycemia, as the main component of the disease, would affect different parts of the body (4). Chronic high blood glucose is the root cause of developing future complications including microvascular (neuropathy, retinopathy and nephropathy) and macrovascular (cardiovascular, peripheral vascular and nervous system) conditions (5). As a result, keeping blood glucose near the normal level is the most important way to prevent subsequent complications.

There is some evidence that 1% decrease in HbA1c level would be associated with 21% reduction in diabetes-related deaths and 12% to 43% reduction in aforementioned conditions (4). Therefore, diabetic patients are advised to maintain their HbA1c at a reasonable level (below or around (6, 7).

Despite the numerous benefits of good glycemic control, large proportions of patients do not reach the target level of HbA1c and remain uncontrolled (7, 8). This is because of the complexity of reasons contributing to poor glycemic control in clinical situation such as disease nature, inadequate or incomplete treatment and care providers’ attitudes (9).

In current study, we analyzed poorly controlled patients’ information by clustering method in order to find more homogenous sub-groups of patients for further scrutinizing our failure to bring their disease under control. This method of grouping helps us to identify characteristics of diabetic patients who are at higher risk of developing complications. Based on these findings, we will be able to plan interventions that are more appropriate for identified groups.

## Methods

This study was conducted based on medical records of diabetic patients admitted to 23 health care centers in four cities of Iran from 2010 to 2014. In 2010, Tehran University of Medical Sciences decided to implement chronic care model (CCM) through health care system in order to improve non-communicable disease care and outcomes (10). For this purpose, 10 centers in south of Tehran, six in Rey, 4 in Eslamshahr and 3 in Yazd were selected.

These centers provide screening services for people who are older than 30 and deliver regular and specific care to patients diagnosed as prediabetic, diabetic, hypertensive, hyperlipidemic, overweight or obese. Diabetic patients are also provided with regular care based on well-developed guidelines and protocols, and their clinical and biochemical data are recorded in data collection software.

All diabetic patients, who participated in the program for 6 months or more and had at least two measurements of HbA_1c_ which the last one was more than eight (11), were considered as poor glycemic control and included in the study.

### Study data

Since physical measurements are recorded in all visits and demographic variables are always kept up to date; the last record in the required biochemical values were present was used to extract the essential parameter for the analysis. These parameters are: 1) Demographic data including age, sex, marital status and educational level; 2) Physical measurements including: systolic blood pressure, diastolic blood pressure, height and weight; 3) Biochemical measurements including blood triglyceride, total cholesterol, LDL, HDL and creatinine. Body mass index (BMI) as one of variables was calculated from division of weight in kilograms by height in meters squared.

### Statistical analysis

Data were analyzed using SPSS ver. 16. According to available data, we performed cluster analysis in order to identify some homogenous groups of patients. For this purpose, the first step after sample selection was definition of some variables describing individuals’ characteristics. Twelve variables as mentioned above, were utilized which were either continuous or categorical. Therefore, the most suitable clustering method for this study was two-step cluster analysis, as this type of cluster analysis has the ability to handle large data and both continuous and categorical variables could be included. Moreover, chi-square and ANOVA were conducted to compare categorical and continuous variables between clusters. In addition, post-hoc tests (Scheffé's tests) were applied to continuous variables. *P-* values less than 0.05 were considered statistically significant.

## Results

Totally 72131 clients have attended our CCM-based centers out of whom 17159 were diagnosed as diabetics. About 2246 out of 7521 patients who were under care for more than six months and had at least two measurements of HbA_1c_ that the last one was more than eight were poorly controlled. About two third of patients were female and the mean age of the sample was 56 yr (range: 26–89). Almost all patients had low to moderate level of education. One-third of patients (36%) had hypertriglyceridemia but only 7% of patients were suffering from hypercholesterolemia ( Table 1 ).

**Table 1:** Sample characteristics

** Variables **	** Category **	** Frequency (%) or Mean(SD) **
Gender	Male	702 (34)
	Female	1385 (66)
Education	Illiterate	843 (40)
	Under high school diploma	1202 (58)
	College education	42 (2)
Marital status	Married	1836 (88)
	Widow	226 (10.8)
	Divorced	16 (0.8)
	Single	9 (0.4)
Age (yr)		56 (10)
BMI (Kg/m^2^)		29 (4.6)
Triglyceride	Normal	790 (38)
N (%)	Borderline	545 (26)
	high	752 (36)
Cholesterol	normal	1397 (67)
N (%)	borderline	550 (26)
	high	140 (7)
Creatinine (Mg/dl)		0.92 (0.2)
Systolic BP (mmHg)		125 (12.3)
Diastolic BP (mmHg)		78 (6.7)
LDL (Mg/dl)		102 (26)
HDL (Mg/dl)		45 (9.5)

Two-step cluster analysis revealed three distinct clusters of 2087 eligible cases. The number of members in each cluster and probabilities of included variables in analysis related to that cluster are described in Table 2 in detail. Eight patients were categorized as outliers, and excluded from these three clusters.

**Table 2:** Demographic, biochemical and clinical characteristics of Clusters’ members

** Variables **		** Cluster 1 **	** Cluster 2 **	** Cluster 3 **	***P* value **
		696 (33)	498 (24)	893 (43)	
Sex, n (%)	Female	0	492 (98.7)	893 (100)	<0.001
	male	696 (100)	6 (1.3)		
Education, n (%)	Illiterate	165 (24)	250 (50)	428 (48)	<0.001
	Under diploma	502 (72)	244 (49)	456 (51)	
	College attainment	29 (4.5)	4 (1)	9 (1)	
Marital status, n (%)	Married	682 (98)	437 (88)	717 (80)	_
	Widow	7 (1)	58 (11.5)	161 (18)	
	Divorced	3 (0.4)	0	6 (1)	
	Single	4 (0.6)	3 (0.5)	9 (1)	
Age	Years	57.4 (10.9)	55.8 (9.3)	55.1 (9.6)	<0.001 ^*^
Mean (SD)					
BMI	Kg/m ^ 2 ^	27.3 (3.8)	29.6 (4.5)	30 (4.8)	<0.001 ^*^
Mean (SD)					
Creatinine	Mg/dl	1.02 (0.22)	0.85 (0.16)	0.86 (0.16)	<0.001 ^*^
LDL	Mg/dl	97.6 (25.8)	127.2 (23)	93.3 (18.5)	<0.001
HDL	Mg/dl	41.5 (8.7)	47.3 (9.1)	46.5 (9.5)	<0.001 ^*^
Systolic BP	mmHg	126 (12)	126 (13.6)	123 (11.6)	<0.001 ^**^
Diastolic BP	mmHg	78.8 (6.6)	78.9 (6.9)	77.1 (6.5)	<0.001 ^**^
Triglyceride, n (%)	Normal	298 (43)	98 (20)	394 (44)	<0.001
	Borderline	170 (24)	113 (23)	262 (30)	
	High	228 (33)	287 (57)	237 (26)	
Cholesterol, n (%)	Normal	529 (76)	0	868 (97)	<0.001
	Borderline	142 (20)	383 (77)	25 (3)	
	High	25 (4)	115 (23)	0	

* no significant difference between clusters 2 & 3

** no significant difference between clusters 1 & 2

Cluster 1 consisted of 696 patients that all of them were men and most of them were moderately educated. They had higher mean age, higher mean creatinine and lower BMI in comparison with two other clusters.

Patients included in cluster 2 (n=498) had the highest probability of experiencing hypertriglyceridemia and borderline cholesterolemia. In addition, the mean value of LDL was higher than its value in members of two other clusters and HDL level was less than what is considered optimal in women. They were more likely to be female and were distributed evenly between illiterate and under high school diploma.

Cluster 3 had the largest number of members (n=893). Members of this cluster were females and, similar to patients in cluster 2, had high average BMI that categorizes them among obese people. About half (44%) had normal range of triglyceride and nearly all of them experienced normal level of cholesterol. LDL mean level was optimal and HDL had the highest level amongst the clusters but there was no significant difference with HDL level in cluster 2.

The results of post-hoc tests (Scheffé's test) revealed that means of continuous variables between each two clusters were significantly different except systolic and diastolic blood pressure between cluster 1 and 2, and age, BMI, HDL and creatinine between cluster 1 and 3 ( Table 3 ).

**Table 3:** Results of Post hoc test’s (Scheffé's test)

** Variables **	** Cluster 1 vs. 2 **		** Cluster 1 vs. 3 **		** Cluster 2 vs. 3 **	
	** Mean difference **	***P* value **	** Mean difference **	***P* value **	** Mean difference **	***P* value **
Age (yr)	1.6	0.02	2.28	<0.001	−1.6	0.47
Systolic BP (mmHg)	−0.3	0.91	3.13	<0.001	3.43	<0.001
Diastolic BP (mmHg)	−0.05	0.992	1.69	<0.001	1.74	<0.001
LDL (mg/dl)	−29.5	<0.001	4.39	0.001	33.9	<0.001
HDL (mg/dl)	−5.79	<0.001	−5	<0.001	0.78	0.3
BMI (Kg/m^2^)	−2.27	<0.001	−2.61	<0.001	−0.34	0.38
Creatinine (mg/dl)	0.18	<0.001	0.17	<0.001	−0.006	0.8

## Discussion

This is the first study trying to explore different sub-types of poor-controlled diabetic patients through cluster analysis method. Cluster analysis is a good way to classify objects; therefore, they become as similar as possible within clusters belonged to and as dissimilar as possible to members of the others (12). This ultimately leads to identification of shared characteristics associated with the specific status like diabetes poor control and could shed light on application of appropriate managements.

Members of cluster 1 were more likely to be men with higher mean age and creatinine in comparison with two other clusters.

Although, increment of creatinine -as a sensitive marker of renal function- may be a sign of renal damage especially in diabetic patients (13), the higher level of this parameter in cluster 1 does not seem to be alarming as mean value is in the normal range. Moreover, men generally have higher level of serum creatinine in comparison with women because of higher skeletal muscle mass (14). *Courtney* states in his paper that male gender, attitudes, and beliefs related to masculinity can be a risk factor for poor health (15). Men have lower tendency to seek help when they face any problem in their lives (16). They seek health-related care with delay when they become sick and use medical care fewer than what is available for them (17). Meanwhile, when they visit their care providers, they ask them fewer questions about disease nature and treatment process than women do (15). Furthermore, their engagement in full-time works and employments affects their health service usage (18). In order to reach better health condition for men, health care providers should deliver appropriate care for them considering their attitudes and beliefs on poor health and sickness. Moreover, establishment of suitable health services in some workplaces may be a good solution to increase service utilization.

Members of clusters 2 and 3 were more likely to be female and obese. The prevalence of obesity among women is higher than that among men in EMRO with approximately 2:1 ratio (19). In this region, nearly half of women experience over-weight and obesity. Numerous studies have demonstrated the obesity as a risk factor for poor glycemic control in diabetic patients (20–22). There is a strong relationship between obesity and insulin resistance (23). In the presence of obesity, insulin binding to fat cell receptors becomes disrupted and sensitivity of receptors decrease consequently (24). In addition, mitochondrial mass and/or activity reduction in obese individuals may contribute to both insulin resistance and beta cell malfunction (25). Furthermore, weight gain is an undesirable and negative consequence of treatment with insulin (26) and therefore, some physicians postpone insulin therapy in obese diabetic patients (27). These factors will probably predispose diabetic patients to poor control condition.

Patients in cluster 2 had higher probability of lipid disturbance including hypercholesterolemia, hypertriglyceridemia, LDL increase and HDL reduction. Diabetic patients, who have concurrent hyperlipidemia, have 5 times more chance of failure in glycemic control than their counterparts without this comorbidity (28). At a closer look, hypercholesterolemia acts as an important factor to poor control in diabetics (29). Therefore, tight control of hyperlipidemia especially high blood cholesterol with dietary and medical treatment must be considered in diabetes particularly in poorly controlled patients.

Aggregation of high triglyceride, low HDL and high blood glucose in the members of cluster 2 was a guide to considering metabolic syndrome (Mets). Third report of National Cholesterol Education Program has mentioned that, when a person has three or more components of a defined set of criteria, he/she can be considered to have metabolic syndrome. These criteria are: TG level more than 150, HDL level less than 50 in women and less than 40 in men, FBS level more than 110, waist circumference more than 88 in women and more than 102 in men and blood pressure more than 130/85(30). In accordance with the literature, metabolic syndrome is associated with improper glycemic control in diabetic patients (31). Mets can be a promoter to insulin resistance and may play a role in deterioration of glycemic control (32). Moreover, Mets are one of the main risk factors for cardiovascular disease (CVD). Women with Mets had about 6-fold and men had about 4-fold increased risk of developing cardiovascular disease in their lives (33). Since diabetes, hypercholesterolemia and obesity are also important risk factors for CVD (34), it is recommended that screening for and management of Mets be considered as a component of diabetic care programs, especially in the presence of obesity and high blood cholesterol to improve diabetes care and outcomes (35).

Illiteracy as an aspect of education was more prominent in clusters 2 and 3 (about half of them). Lower level of literacy is associated with lower level of knowledge about diabetes nature and may have a role in poor control of the disease (36). Literacy can affect personal knowledge about heath, disease characteristics, outcomes and individuals with lower ability to read, experience more unfavorable outcomes that are 1.5 to 3 times that of individuals with higher ability. Illiterate people encounter more problems not only in reading drug labels, but also in communicating with their physicians and understanding their medical orders (37). On the other hand, illiterates are more likely to be uninsured or suffer from poverty (37, 38). In order to deal with mentioned problems, diabetic patients should be instructed in their disease and provided with helpful and suitable educational materials. As well, health care providers should be empowered with health communication skills in order to improve patient-physician relationship especially in the case of illiteracy (39).

## Conclusion

Masculinity would play the main role in diabetes control. Moreover, aggregation of obesity, illiteracy and/or dyslipidemia in diabetic women pre-dispose them to poor control condition.

## Ethical consideration

Ethical issues (Including plagiarism, informed consent, misconduct, data fabrication and/or falsification, double publication and/or submission, redundancy, etc.) have been completely observed by the author
